# Combining genetic and non-genetic risk factors to predict disease, and reporting the screening performance of risk models

**DOI:** 10.1177/09691413231196124

**Published:** 2023-08-24

**Authors:** Robert Old

**Affiliations:** 2707 University of Warwick, Coventry, UK

In the span of a generation, medical genetics has been transformed by genome analysis. The hope now is that DNA analysis can significantly account for the heritable component of common diseases such as heart attack, stroke and some cancers and can, therefore, contribute usefully to screening programmes. But screening based on risk factors other than age is difficult. Predicting disease rather than simply detecting its early signs is the challenge.

Mutations in single major effect genes, such as BRCA1 and BRCA2, confer high risk and if a remedy is available may merit consideration of screening programmes. But, generally, most cases of disease in most populations are not caused by mutations of major or intermediate-risk genes.

The polygenic contribution to disease is approachable either by direct DNA sequence analysis of the genome or more economically by microarray hybridization. Genome-wide association studies (GWAS) establish the link between typically hundreds, up to as many as millions, of single nucleotide polymorphisms (SNPs) and genetic risk. The small risks associated with each of the unfavourable SNP variants are assumed to combine multiplicatively and their joint effect can be summarized as a polygenic risk score (PRS) for any individual and a particular disease.

Wald and I have shown^1^ that although a PRS can provide information on the etiology of a disease, to date PRSs have minimal efficacy in screening for disease, notably heart attack, stroke and certain cancers; these being the diseases where most PRS studies have been carried out. In practice, clinicians are interested in knowing the detection rate (DR, also known as sensitivity) and false-positive rate (FPR, 1 – specificity) of a proposed screening test. Where research reports give metrics such as the odds ratio for high and low centile groups of the predicted risk distribution there is a published Risk Screen Converter (freely available at https://www.medicalscreeningsociety.com/rsc.asp on the Medical Screening Society website) from which the values of the two most relevant measures of screening performance can be read. These show that, on the basis of what has been published, the PRS-based models do not in practice provide useful screening performance.

In an attempt to improve screening further, more comprehensive risk models have been devised which include both genetic and non-genetic risk factors. This substantial research task has been undertaken for breast cancer and prostate cancer. Taking the breast cancer example,^2^ the new methodology incorporates some variants of single genes with major (BRCA1, BRCA2) and intermediate effects (PALB2, CHEK2, ATM), 313 SNPs, other familial risks, and known questionnaire-based risk factors (age at menarche, menopause, first live birth, hormonal risk factors, mammographic density where known). Applying the model to the UK, the authors reported that the lowest centile of the risk distribution had a predicted lifetime risk of 2.8%, and 30.6% (about an eleven-fold ratio) for the highest centile, which the Risk Screening Converter shows is equivalent to a DR of 12% for a 5% FPR.

For prostate cancer,^3^ for the UK population after a 10-year observation period, the 50% of men with the higher predicted risks captured 86.3% of the observed prostate cancer. Construction of a 2  ×  2 table in which 3.85% (0.863  ×  4.46% incidence in 10 years) are in the 50% category of screen-positive and detected, and 46.15% are screen-positive and unaffected, shows these data are equivalent to a DR of 86.3% for an impracticably high FPR of 48.3%.

Despite the substantial effort put into deriving complex models that combine the risk factors, the picture is one in which the great majority of people who develop disease are without particularly high predicted risk, making the screening performance insufficient to be worthwhile.

Some conclusions and suggestions may be of value. First, combined genetic and non-genetic risk models that have been devised to date are too poor for disease prediction and population screening. Polygenic risk scores that have been devised so far incorporate typically hundreds or many thousands of SNPs. Adding more SNPs is unlikely to improve prediction markedly. The law of diminishing returns applies to GWAS studies. But there is a continuing problem of ‘missing heritability’, the genetic risk factors do not capture all the genetic risk. This may be improved by including more variants of major and intermediate risk genes as further research clarifies ‘variants of unknown significance’. In addition, these combined risk prediction models exclude epigenetic effects, such as the role of DNA methylation in gene function.

Second, it would be helpful if those who derive models and assess their use in screening would express the performance of their model in terms of DR, FPR and OAPR (odds of being affected given a positive result, which is related to positive predictive value, PPV). For example, the concordance index (C-index) may be a useful statistic for comparing models, but this measure combines (and obscures) DR and FPR.

Third, where the screening performance of a model is insufficient as a stand-alone method, authors often suggest its use for risk stratification. This cannot transform a poor screening test into a good one. The utility must still derive from the DR and FPR of the proposed strata of risk, plus the cost and complexity of implementing the programme.

Fourth, genome analysis can be performed on an individual once in a lifetime, perhaps in childhood, and used to assess genetic risk factors for a panel of several diseases according to prediction models for each disease. Proponents of this idea should, however, recognize that there will be an FPR for each disease in the panel, and for an individual person these are additive. A panel of eight diseases each with an FPR of 5% results in 40% of the screened population being screen (false) positive, and a challenge for any healthcare system.
